# Comparison of the effects of different foam rolling durations on knee extensors function

**DOI:** 10.5114/biolsport.2024.131820

**Published:** 2023-10-17

**Authors:** Kazuki Kasahara, Andreas Konrad, Riku Yoshida, Yuta Murakami, Ryoma Koizumi, Ewan Thomas, David G Behm, Masatoshi Nakamura

**Affiliations:** 1 Institute for Human Movement and Medical Sciences, Niigata University of Health and Welfare, Niigata, Japan; 2 Institute of Human Movement Science, Sport and Health, University of Graz, Graz, Austria; 3 Department of Physical Therapy, Niigata University of Health and Welfare, Niigata, Japan; 4 Sport and Exercise Sciences Research Unit, Department of Psychology, Educational Science and Human Movement, University of Palermo, Palermo, Italy; 5 School of Human Kinetics and Recreation, Memorial University of Newfoundland, St. John’s, Newfoundland and Labrador, Canada; 6 Faculty of Rehabilitation Sciences, Nishi Kyushu University, 4490-9 Ozaki, Kanzaki, Saga, 842-8585, Japan

**Keywords:** Self-myofascial release, Roller massage, Range of motion, Flexibility, Maximal voluntary concentric contraction, Tissue hardness, Prolonged effect

## Abstract

Foam rolling (FR) intervention has recently attracted attention in sports and rehabilitation settings. However, the effects of FR using different rolling durations have not been fully clarified. Thus, this study focused on FR durations and examined the acute and prolonged (i.e., 20-min; 40-min, 60-min) effects of different FR intervention durations on maximal voluntary concentric contractions (MVC-CON), knee flexion range of motion (ROM), pain pressure threshold (PPT), and tissue hardness. The participants were 10 male university students (22.5 ± 1.0 years), and the target muscles were the dominant leg knee extensors. Three sets of 60-seconds FR interventions were performed in the randomized crossover trials in each condition. The three intervention conditions were fast (1 rolling/2 s, 30-repetition × 3 sets, 90 repetitions), medium (1 rolling/6 s, 10-repetition × 3 sets, 30 repetitions), and slow speed (1 rolling/12 s, 5-repetition × 3 sets, 15 repetitions). Before as well as immediately, 20-min, 40-min, and 60-min after the interventions, MVC-CON, ROM PPT, and tissue hardness were measured. The results showed no interaction effect in the acute effect but a main effect of time for all variables (p < 0.05). Also, no interaction was observed in prolonged effect, but main effects of time were observed in knee flexion ROM, PPT, and tissue hardness (p < 0.01) but not for MVC-CON. Post-hoc tests showed significant PPT (p < 0.05) and knee flexion ROM (p < 0.01) increases up to 20- and 60-minutes respectively after all interventions. Tissue hardness was significantly (p < 0.01) decreased up to 60-minutes after all interventions. This study showed that the FR intervention changed ROM, PPT, tissue hardness, and MVC-CON regardless of rolling duration and that the effects persisted up to 20–60 minutes.

## INTRODUCTION

Previous studies showed that foam rolling (FR) could increase the range of motion (ROM) without decreasing muscle strength or performance [1–3]. Furthermore, FR could decrease tissue hardness [1]. For these reasons, FR has attracted attention as a warm-up method. In addition, previous studies showed FR interventions could decrease pain associated with delayed onset muscle soreness [4–6]. Moreover, FR interventions could decrease pain in post-operative patients [7, 8]. Therefore, FR is applied in both sports and rehabilitation.

Although many previous studies have shown the effectiveness of FR interventions, detailed data on the use of FR are sparse. A previous study examining optimal FR intensities showed that ROM increases were possible regardless of low (rating of perceived pain (RPP): 3.9/10), moderate (RPP: 6.2/10), or high (RPP: 8.2/10) rolling pressures were applied without negatively affecting muscle strength or performance [9]. Soft, medium, and hard (EVA foam) FR densities all showed increased knee flexion ROM and PPT regardless of FR densities [10]. Moreover, the minimum duration of FR intervention to increase ROM might differ between target muscles. Nakamura et al. [23] reported that FR of the calf muscles for at least 90-seconds could be expected to increase dorsiflexion ROM. On the other hand, Sullivan et al. [21] reported that a 5-second FR intervention on the knee flexors significantly increased sit and reach ROM. In FR intervention, individual rolling duration (speed) is considered an essential factor in addition to intensity (pressure), total FR session time, and density. Still, only a few previous studies have focused on the FR durations. Wilke et al. [11] examined the effects of different intervention duration on the anterior thigh with an intervention time of 45 seconds × 4 sets and an intervention intensity of Numerical Rating Scale (NRS) 6–7. The three rolling duration conditions were compared: a fast intervention condition of 1 roll/2-seconds, a slow intervention condition of 1 roll/10-seconds, and a control condition. The results showed no increase in knee flexion ROM in any conditions. On the other hand, they reported a significant decrease in tissue stiffness after 5- and 10-minutes in the fast intervention condition and after 10-minutes in the slow intervention. A commentary by Behm et al. [13] suggested that a rolling duration of 2–4-seconds per direction is optimal for increasing ROM. However, they used regression equations from a prior study data to predict optimal responses rather than conducting a research study to directly compare rolling durations (speeds). However, to our knowledge, the effects of different rolling durations in FR interventions have not been fully investigated. In addition, the previous study by Wilke et al. [11] only examined the effects of FR at different durations up to 10-minutes post-rolling. In our previous study [1], a 180-second FR intervention on knee extensors significantly increased ROM at least 30-minutes after the intervention. However, the duration of increased ROM after FR is unknown, and hence, studies applying longer testing periods than 30 minutes are needed to investigate the prolonged effects of FR. Therefore, this study’s objectives were the following: The first was to compare and examine the acute effects of different FR intervention durations (speeds) on maximal voluntary concentric contractions (MVC-CON), range of motion (ROM), pain pressure threshold (PPT), and tissue hardness of the knee extensors. The second was to compare the prolonged effect of different FR intervention durations (speeds). Behm et al. [13] reported that the effect of FR on increasing ROM was greater at an intervention duration of 2–4 seconds per direction. In addition, our previous study [1] found a significant increase in ROM at an intervention duration of 1 second per direction. It has also been reported that FR does not decrease muscle strength and performance [12, 13]. Therefore, in this study, we hypothesized that the effect of increasing ROM is larger the fast and smaller the later. In addition, we considered that muscle strength and performance would not change regardless of the FR duration (speed).

## MATERIALS AND METHODS

### Experimental set-up

**FIG. 1:**
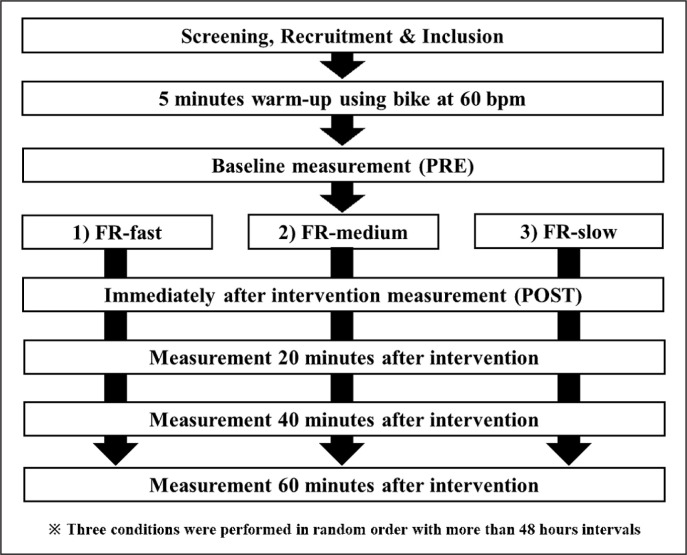
The experimental set-up for foam rolling (FR) intervention with three durations: FR-Fast, FR-Medium, and FR-Slow. For FRFast, FR-Medium, and FR-Slow, rolling interventions were performed from proximal to distal and back to proximal of the dominant (preferred to kick a ball) knee extensors in 2-, 6-, and 12-seconds, respectively. One set was 60-seconds for each condition, and three sets were performed (total of 180-seconds). The measured parameters were maximal voluntary concentric contraction torque, knee flexion range of motion, pain pressure threshold and tissue hardness in all time intervals.

A repeated randomized measures experimental design was used to compare differences in the duration of FR intervention. The participants were instructed to visit the laboratory three times with a ≥ 48 h break. They were exposed to the following three conditions: FR-Fast, FR-Medium, and FR-Slow in a random order (Figure 1). For FR-Fast, FR-Medium, and FR-Slow, rolling interventions were performed from proximal to distal and back to proximal of the dominant (preferred to kick a ball) knee extensors in 2-, 6-, and 12-seconds, respectively. One set was 60-seconds for each condition, and three sets were performed (total of 180-seconds). The measurement periods were before the intervention (PRE), immediately after (POST), 20-, 40-, and 60-minutes after the intervention. The measurements were tissue hardness, PPT, knee flexion ROM, and MVC-CON, and these were assessed in this order. Since knee flexion ROM and MVCCOM measurements may influence PPT and tissue hardness measurements, measurements were performed in this order.

### Participants

Ten healthy, recreationally active males were enrolled (mean ± SD: age, 22.5 ± 1.0 years; height, 170.1 ± 3.5 cm; weight, 69.8 ± 8.7 kg). The participants completed the three conditions described above in random order. Individuals with a history of neuromuscular disease and musculoskeletal injury involving the lower extremities were excluded. The required sample size for a repeated-measures two-way analysis of variance (ANOVA) (effect size = 0.25 [large when considering interaction effects for 2-way ANOVAs], αerror = 0.05, and power = 0.80) based on our previous study’s ROM results [14] using G* power 3.1 software (Heinrich Heine University, Dusseldorf, Germany) was more than eight participants.

For the study, participants were fully informed about the procedure and aims, after which they provided written informed consent. The study complied with the requirements of the Declaration of Helsinki and was approved by the Ethics Committee of the Niigata University of Health and Welfare, Niigata, Japan (Procedure #18615).

### Outcome assessment

#### Knee flexion ROM

Each participant was placed in a side-lying position on a massage bed with the hips as well as the knee of the non-dominant leg flexed at 90° to prevent pelvic movements [1, 15]. A licensed physical therapist, the investigator, brought the dominant leg to full knee flexion with the hip joint in a neutral position. A goniometer (MMI universal goniometer Todai 300 mm, Muranaka Medical Instruments, Co., Ltd., Osaka, Japan) was used to measure knee flexion. ROM was measured three times in each measurement period, and the average value at each measurement period was used for analysis.

### Pain pressure threshold (PPT)

PPT measurements were conducted in the supine position using an algometer (NUTONE TAM-22(BT10); TRY-ALL, Chiba, Japan). The measurement location was set at the midway point of the distance between the anterior superior iliac spine and the dominant side’s superior border of the patella for the rectus femoris muscle. With continuously increasing pressure, the soft tissue in the measurement area was compressed with the metal rod of the algometer. The participants were instructed to immediately press a trigger when pain, rather than just pressure, was experienced. The value read from the device at this time point (kilograms per square centimeter) corresponded to the PPT. In each condition, PPT was measured three times at each measurement period, and the mean value at each measurement period was used for further analysis.

### Tissue hardness

Tissue hardness was measured using a portable tissue hardness meter (NEUTONE TDM-N1; TRY-ALL Corp., Chiba, Japan). The participant’s measurement position and posture were similar to PPT measurements. This tissue hardness meter measured the penetration distance until a 14.71 N (1.5 kgf) pressure was reached [16]. The participants were instructed to relax while tissue hardness was measured three times at each measurement period, and the mean value at each measurement period was used for further analysis.

### Maximal Voluntary Concentric Contractions (MVC-CON)

In accordance with previous studies [17], MVC-CON of the dominant leg’s knee extensors was measured at an angular velocity of 60°.s^−1^ between 20° and 110° knee flexion using an isokinetic dynamometer (BIODEX System 3.0, Biodex Medical System Inc. Shirley, NY, USA). The participants sat on the dynamometer chair adopting an 80° hip flexion angle, with adjusted Velcro straps fixed over the exercised limb’s trunk, pelvis, and thigh. Three trials were performed at each measurement period, and the highest value was analyzed. In all trials, strong verbal encouragement was given to elicit maximum effort.

### Foam rolling (FR) Intervention

A physical therapist instructed the participants how to use the foam roller (Stretch Roll SR-002, Dream Factory, Umeda, Japan). For familiarization, they were allowed to practice using the foam roller three to five times on the non-dominant leg (non-intervention leg) immediately before the FR intervention to verify that the participants were able to perform the FR intervention at the specified duration (speed) and location. FR was performed using three sets of 60-seconds with a 30-seconds rest between sets. One cycle of FR was defined as one distal rolling movement followed by one proximal rolling movement. FR-fast was performed in 2-seconds (30 repetitions × 3 sets, 90 repetitions), FR-medium in 6-seconds (10 repetitions × 3 sets, 30 repetitions), and FR-slow in 12-seconds (5 repetitions × 3 sets, 15 repetitions) for one cycle. A metronome (Smart Metronome; Tomohiro Ihara, Japan) was used for control. The participants were asked to place as much body mass on the roller as tolerable. All FR interventions were supervised by one well-trained physical therapist.

### Statistical analysis

SPSS (version 28.0; IBM Corp., Armonk, NY, USA) was used for the statistical analysis. We calculated the coefficient of variation (CV) and intraclass correlation coefficient (ICC) from PRE data in three conditions to check the test-retest reliability. To verify the consistency of PRE values, PRE values were tested among all conditions using a one-way ANOVA. To clarify the difference with the acute effects of different rolling duration, two-way 2 × 3 repeated measures ANOVA using two factors (test time [PRE vs. POST] and condition [FR-fast vs. FR-medium vs. FR-slow]) was analyzed for interactions and main effects. For the prolonged effect, a two-way 3 × 3 repeated measures ANOVA using two factors (test duration [PRE vs 20 min vs 40 min vs 60 min] and condition [FR-fast vs FR-medium vs FR-slow]) was analyzed for interactions and main effects. Classification of effect size (ES) was set where $\eta_{\text{p}}^{2}$ < 0.01 was considered small, 0.02–0.1 was considered medium, and more than 0.1 was considered to be a large effect size [18]. As post-hoc tests, a paired t-test with Bonferroni correction was used for the acute effect, and a multiple comparison test with Bonferroni correction was used for the prolonged effect. Additionally, we calculated the Cohen’s d as effect size between PRE and POST, 20-, 40-, 60-minutes in each condition, respectively, distinguishing trivial (d = 0–0.19), small (d = 0.20 to 0.49), medium (0.50 to 0.79) or large (≥ 0.80 or higher) effects [18]. The significance level was set to 5%, and all the results are shown as mean ± SD.

## RESULTS

### Comparison between PRE values among the three conditions

There were no significant differences in all PRE variables between the three conditions. The CVs of measurements for MVC-CON, knee ROM, PPT, and tissue hardness were 6.0 ± 2.8%, 1.5 ± 1.2%, 12.7 ± 7.6%, and 9.6 ± 4.3%, respectively, and the ICC (1,3) for measurements were 0.929, 0.563, 0.735, and 0.854, respectively.

### Acute effects in MVC-CON, knee flexion ROM, PPT, and tissue hardness

Table 1 shows the changes in MVC-CON, knee flexion ROM, PPT, and tissue hardness, before (PRE) and immediately following (POST) the three durations of FR intervention. There were no significant interaction effects for all variables (MVC-CON: F = 0.1, p = 0.93, $\eta_{\text{p}}^{2}$ = 0.01, knee flexion ROM: F = 0.3, p = 0.71, $\eta_{\text{p}}^{2}$ = 0.03, PPT: F = 0.2, p = 0.84, $\eta_{\text{p}}^{2}$ = 0.01, tissue hardness: F = 0.4, p = 0.70, $\eta_{\text{p}}^{2}$ = 0.03). However, there were significant main effects of test time with all variables (MVC-CON: F = 5.7, p < 0.05, $\eta_{\text{p}}^{2}$ = 2 0.18, knee flexion ROM: F = 87.2, p < 0.01, $\eta_{\text{p}}^{2}$ = 0.76, PPT: F = 36.5, p < 0.01, $\eta_{\text{p}}^{2}$ = 0.58, tissue hardness: F = 44.9, p < 0.01, $\eta_{\text{p}}^{2}$ = 0.62). The post-hoc test results showed that MVC-CON (p < 0.05), knee flexion ROM (p < 0.01), PPT (p < 0.01) were significantly higher at POST and tissue hardness (p < 0.01) was lower at POST.

**TABLE 1:** The acute changes (mean ± SD) in MVC-CON torques, knee flexion range of motion (ROM), pain pressure threshold (PPT), and tissue hardness before (PRE) and immediately after (POST) the intervention.

	FR-fast		FR-medium		FR-slow	

	PRE	POST	PRE	POST	PRE	POST
MVC-CON (Nm)	184.4 ± 28.4	192.5 ± 26.2^*^	189.2 ± 40.5	194.9 ± 27.5^*^	188.1 ± 22.3	194.1 ± 23.0^*^
	d = 0.30		d = 0.17		d = 0.27	

Knee flexion ROM (deg)	137.5 ± 2.1	140.5 ± 2.6^*^	137.1 ± 4.7	140.2 ± 4.0^*^	137.7 ± 3.9	141.3 ± 4.0^*^
	d = 1.27		d = 0.72		d = 0.92	

PPT (kg)	4.2 ± 1.2	5.1 ± 2.1^*^	3.9 ± 0.8	5.0 ± 1.4^*^	3.5 ± 0.8	4.4 ± 1.1^*^
	d = 0.54		d = 1.01		d = 0.92	

Tissue hardness (N)	18.3 ± 4.1	16.0 ± 3.4^*^	17.9 ± 3.8	16.2 ± 3.0^*^	19.3 ± 3.6	17.5 ± 4.2^*^
	d = -0.61		d = -0.51		d = -0.47	

* : Significant difference (p < 0.05) from PRE.

### Prolonged effects in MVC-CON, knee flexion ROM, PPT, and tissue hardness

Table 2 illustrates MVC-CON, knee flexion ROM, PPT, and tissue hardness values, PRE, 20-min, 40-min, and 60-min after three durations of FR intervention. There were no significant interaction effects for all variables (MVC-CON: F = 0.3, p = 0.95, $\eta_{\text{p}}^{2}$ = 0.02, knee flexion ROM: F = 1.25, p = 0.29, $\eta_{\text{p}}^{2}$ = 0.09, PPT: F = 0.5, p = 0.82, $\eta_{\text{p}}^{2}$ = 0.04, tissue hardness: F = 0.3, p = 0.92, $\eta_{\text{p}}^{2}$ = 0.02). However, there were significant main effects of test time with knee flexion ROM, PPT, and tissue hardness (knee flexion ROM: F = 58.3, p < 0.01, $\eta_{\text{p}}^{2}$ = 0.68, PPT: F = 6.4, p < 0.01, $\eta_{\text{p}}^{2}$ = 0.19, tissue hardness: F = 22.6, p < 0.01, $\eta_{\text{p}}^{2}$ = 0.46). There were no prolonged effects with MVC-CON. The post-hoc tests showed that knee flexion ROM (p < 0.01), PPT (p < 0.01) were significantly higher at POST and tissue hardness (p < 0.01) was lower at POST. Post-hoc test results showed that knee flexion ROM was significantly (p < 0.01) higher 20-, 40-, and 60-minutes after the intervention compared with PRE. The values at 20-minutes were significantly (p < 0.01) higher than at 40- and 60-minutes, and the values at 40-minutes were significantly (p < 0.01) higher than at 60-minutes. PPT was significantly (p < 0.05) higher at 20-minutes compared to PRE. Tissue hardness was significantly (p < 0.01) lower 20-, 40-, and 60-minutes after the intervention compared with PRE. The values at 60-minutes were significantly (p < 0.01) higher than at 20- and 40-minutes.

**TABLE 2:** The prolonged changes (mean ± SD) in maximal voluntary concentric contraction (MVC-CON) torques, knee flexion range of motion (ROM), pain pressure threshold (PPT), tissue hardness PRE and after 20-, 40-, and 60-minutes after the interventions.

	FR-fast				FR-medium				FR-slow			

	PRE	20 min	40 min	60 min	PRE	20 min	40 min	60 min	PRE	20 min	40 min	60 min
MVC-CON (Nm)	184.4 ± 28.4	192.5 ± 26.2	185.6 ± 25.9	183.5 ± 22.2	189.2 ± 40.5	188.8 ± 32.4	187.0 ± 33.2	182.5 ± 30.5	188.1 ± 2.3	192.8 ± 20.7	189.9 ± 20.6	188.1 ± 17.0

	d =	0.19	0.04	-0.04	d =	-0.01	-0.06	-0.19	d =	0.22	0.08	0.00

Knee flexion ROM (deg)	137.5 ± 2.1	139.7 ± 2.7*	138.7 ± 2.5*†	138.4 ± 2.3*†‡	137.1 ± 4.7	139.0 ± 4.4*	138.3 ± 4.7*†	137.5 ± 4.7*†‡	137.7 ± 3.9	140.3 ± 4.2*	138.6 ± 3.7*†	138.2 ± 3.6*†‡

	d =	0.91	0.52	0.39	d =	0.42	0.26	0.09	d =	0.64	0.24	0.12

PPT (kg)	4.2 ± 1.2	5.1 ± 2.1*	4.7 ± 1.9	4.6 ± 1.8	3.9 ± 0.8	4.4 ± 1.4*	4.2 ± 1.2	3.8 ± 0.7	3.5 ± 0.8	4.0 ± 1.2*	4.0 ± 1.2	3.9 ± 1.1

	d =	0.30	0.25	0.05	d =	0.39	0.26	-0.13	d =	0.49	0.44	0.37

Tissue hardness (N)	18.3 ± 4.1	16.8 ± 3.3*	16.7 ± 3.5*	17.2 ± 3.8*†‡	17.9 ± 3.8	16.3 ± 3.3*	16.4 ± 3.0*	17.2 ± 3.2*†‡	19.3 ± 3.6	17.5 ± 3.9*	17.8 ± 3.4*	18.7 ± 3.5*†‡

	d =	-0.41	-0.42	-0.27	d =	-0.45	-0.46	-0.22	d =	-0.49	-0.43	-0.18

* : Significant difference (p < 0.05) from PRE;

† : Significant difference (p < 0.05) from 20 minutes after the intervention;

‡ : Significant difference (p < 0.05) from 40 minutes after the intervention.

## DISCUSSION

The positive effects of an acute bout of FR were not duration specific (from 2 to 12 seconds per rolling direction). This study shows that 180-seconds of FR increased ROM, PPT, MVC-CON and decreased tissue hardness immediately post-test but there were no prolonged MVC-CON effects (20–60-minutes post-intervention). However, PPT increased for up to 20-minutes whereas both ROM increased, and tissue hardness decreased for up to 60-minutes after the FR intervention regardless of rolling duration (rolling speeds).

FR has been reported to be effective in increasing ROM [3, 13, 19]. The lack of difference in effectiveness with different FR durations (speeds), support the results of Wilke et al. [11]. However, while knee flexion ROM did not significantly change after 45 seconds × 4 sets of FR in the Wilke et al. study, the present study showed a significant increase in knee flexion ROM at all measured time points. This result must be interpreted through a lens of a moderate (ICC: 0.563) reliability coefficient [20]. This discrepancy may be due to differences in knee flexion ROM measurements, where Wilke et al. [11] employed active knee flexion measurements, this study used passive knee flexion measurements. The influence of FR on the difference between active and passive ROM measurement needs further investigation. Furthermore, whereas individuals seem to focus on or emphasize hip flexion (hamstrings) flexibility, less emphasis and time is typically placed on knee flexion (quadriceps) flexibility. Hence, this lower familiarization with knee flexion ROM may have contributed to the moderate reliability values and the increasing ROM with successive ROM tests at all time points. Still, Wilke et al. [11] reported a significant reduction in tissue stiffness after 5- and 10-minutes of FR in the fast speed condition and only after 10-minutes in the slow speed condition suggesting that the FR effect may be greater with the fast versus slow speed conditions. Behm et al. [13] in their commentary recommended a rolling time of 2–4 seconds per direction to increase ROM. In our previous study [1], FR for 180-s increased ROM up to 30-minutes with FR durations of 1-second per direction. However, in the present study, a significant increase in ROM was also observed in the slow duration condition (12-seconds per 1 roll). Wilke et al. [11] also stated that their study examined acute effects and that prolonged effects need to be examined. In this study, the prolonged effect was examined up to 60 minutes after the intervention, and as with the acute effect, there were no differences between the groups. Although the total duration of the FR intervention in Wilke et al. [11] and the present study were identical, differences in the intervention duration per set and the number of sets might cause differences in the effects of intervention duration. Furthermore, the commentary by Behm et al. [13] recommends 30–120 seconds × 1–3 sets of FR intervention when aiming to increase ROM. Moreover, FR has been suggested to have a volumeresponse relationship [5, 21]. This suggests that increasing the intervention time may have different effects depending on the intervention duration (speed).

In this study, PPT was significantly increased up to 20-minutes, and knee flexion ROM was significantly increased up to 60-minutes. Previous studies have suggested that increases in stretch tolerance (pain sensation) are involved with the increase in ROM after FR and vibration foam rolling interventions [19, 22–24]. FR may reduce pain by activating either neural-gating mechanisms [25, 26] or releasing endorphins and enkephalins as theorized with the diffuse noxious inhibitory control mechanism [27]. In addition, there are reports of increased PPT after FR intervention [1, 28–30]. Although the detailed relationship between increased PPT and increased ROM is not clear, it is possible that FR intervention altered stretch tolerance (pain sensation), resulting in increased ROM. However, ROM remained elevated for 60-minutes, hence, increased pain or stretch tolerance cannot be the primary or sole factor in this increase.

Tissue hardness was significantly decreased up to 60-minutes after the intervention regardless of rolling duration, suggesting that the decrease in tissue hardness may be involved in the maintenance of increased ROM. A systematic review and meta-analysis [31] reported that FR intervention decreases tissue hardness in the quadriceps muscle. FR may decrease tissue hardness through thixotropic changes [32] and increase tissue perfusion [33]. The same mechanism is likely responsible for the significant decrease in the present study.

In this study, regardless of FR duration, MVC-CON was increased immediately following the FR intervention. However, there were no significant prolonged (20–60-minutes post-intervention) changes in MVC-CON regardless of FR duration. A meta-analysis [34] reported that FR did not affect muscle strength or performance. The results of the present study are consistent with a systematic review and meta-analysis by Glänzel et al. [31], suggesting that FR of the knee extensors can significantly increases MVC-CON. The mechanism of FR-induced increase in muscle strength is thought to be due to an increase in local blood flow. Warm-up effects such as increased blood flow are thought to increase muscle temperature and induce nitric oxide release [33, 35] promoting vasodilation and phosphate creatine replenishment. In addition, the thixotropic effect of FR may allow for restful movement and that the decrease in pain sensitivity may allow for higher contractile strength. The effects of FR interventions on muscle strength and performance have not been consistently reported, and much further study is needed.

This study has several limitations: first, the maximum prolonged effect of the FR intervention is unknown. Secondly, since this study was conducted on healthy male university students, it is not known whether the same effects can be obtained on athletes or female subjects. Third, the order of measurements in this study was not randomized; thus, the results may differ depending on the order of the measurements. However, since the order of the measurements was the same before and after the interventions, a potential effect of the order is likely negligible. The fourth limitation is the intervention method. In this study, FR was practiced on the non-dominant side. This may have caused a crossover effect. However, due to the short intervention time and the practice sessions that were standardized for all conditions, the effect of the practice sessions on this study was small. Furthermore, crossover effects or non-local muscle effects have been reported to be trivial when single discrete actions are observed [36]. Fifth, this study examined the effects of different FR intervention durations, but the effects compared to stretching are unknown. Kasahara et al. [17] showed that combined FR and stretching interventions had a cumulative effect, while Yuan et al. [37] did not. Therefore, future studies are needed to examine different combinations of rolling duration and stretching intervention to construct an effective protocol.

### Practical implications

As a pre-exercise warm-up, short-term, i.e., 30-second FR or VFR intervention is recommended when the goal is to increase ROM while maintaining muscle strength. Also, this beneficial effect was prolonged for 10 or 15 minutes after interventions. Also, in short-term interventions, vibration function does not necessarily need to be added to FR as a warm-up routine.

## CONCLUSIONS

When using FR intervention for warm-up, rolling duration (speed) has no effect when the goal is to immediately increase ROM, PPT, and MVC-CON torque and decrease tissue hardness. FR intervention can be expected to increase ROM and decrease tissue hardness up to 60 minutes after FR intervention, regardless of rolling duration (speed), so it can be incorporated into warm-up routines. It is possible to adjust the intervention rate for each subject based on the results of this study, which is important information because it indicates that FR may be applicable in many fields.
